# Retraction: Pulmonary Embolism in COVID-19 Patients: A Retrospective Case-Control Study

**DOI:** 10.7759/cureus.r74

**Published:** 2023-06-26

**Authors:** Asia H Ghawas, Abdulrahman S Althobaiti, Qusai A Almuslem, Mohammed H Bin Nasif, Faisal F Algharbi, Reem A Alshehri, Yousef K Al Gethami, Khaled W Altowayan, Fahad K Alzahrani, Amal A Suwaylih, Abdullah S Alwadai, Abdulmajeed M Badawi, Malak Alshammari

**Affiliations:** 1 General Practice, Dhurma General Hospital, Dhurma, SAU; 2 General Practice, Al Faisaliah Primary Health Care Center, Dammam, SAU; 3 General Practice, Al Naeriyah Hospital, Dammam, SAU; 4 College of Medicine, King Saud University, Riyadh, SAU; 5 College of Medicine, King Khalid University Hospital, Riyadh, SAU; 6 College of Medicine, King Abdulaziz University, Jeddah, SAU; 7 College of Medicine, Taif University, Al-Taif, SAU; 8 College of Medicine, Hadhramout University, Al-Mukalla, YEM; 9 College of Medicine, King Khalid University, Abha, SAU; 10 College of Medicine, Imam Abdulrahman Bin Faisal University, Dammam, SAU

This article has been retracted due to concerns regarding the provenance of the article, as multiple authors have contacted the journal to deny authorship of the article. This article was submitted and subsequently published purportedly as an effort coordinated by Imam Abdulrahman Bin Faisal University to ensure all medical interns publish at least one peer-reviewed article in order to qualify for enrollment in a postgraduate residency program as stipulated by The Saudi Commission for Health Specialties (SCFHS).

Despite the journal's repeated inquiries, both Imam Abdulrahman Bin Faisal University and The Saudi Commission for Health Specialties have failed to respond to numerous communications requesting additional information regarding these allegations. Given that multiple authors have now come forward stating they were not involved in this study, we have made the decision to retract the article despite the lack of formal investigation by Imam Abdulrahman Bin Faisal University and The Saudi Commission for Health Specialties (SCFHS).

